# Genetic Diversity of O-Antigens in *Hafnia alvei* and the Development of a Suspension Array for Serotype Detection

**DOI:** 10.1371/journal.pone.0155115

**Published:** 2016-05-12

**Authors:** Zhifeng Duan, Tomasz Niedziela, Czeslaw Lugowski, Boyang Cao, Tianwei Wang, Lingling Xu, Baopeng Yang, Bin Liu, Lei Wang

**Affiliations:** 1 Key Laboratory of Molecular Microbiology and Technology of the Ministry of Education, TEDA College, Nankai University, Tianjin, P. R. China; 2 TEDA Institute of Biological Sciences and Biotechnology, Nankai University, Tianjin, P. R. China; 3 Tianjin Research Center for Functional Genomics and Biochips, TEDA College, Nankai University, Tianjin, P. R. China; 4 Tianjin Key Laboratory of Microbial Functional Genomics, TEDA College, Nankai University, Tianjin, P. R. China; 5 Hirszfeld Institute of Immunology and Experimental Therapy, Polish Academy of Sciences, Wroclaw, Poland; 6 Department of Biotechnology and Molecular Biology, University of Opole, Opole, Poland; The Pennsylvania State University, UNITED STATES

## Abstract

*Hafnia alvei* is a facultative and rod-shaped gram-negative bacterium that belongs to the *Enterobacteriaceae* family. Although it has been more than 50 years since the genus was identified, very little is known about variations among *Hafnia* species. Diversity in O-antigens (O-polysaccharide, OPS) is thought to be a major factor in bacterial adaptation to different hosts and situations and variability in the environment. Antigenic variation is also an important factor in pathogenicity that has been used to define clones within a number of species. The genes that are required to synthesize OPS are always clustered within the bacterial chromosome. A serotyping scheme including 39 O-serotypes has been proposed for *H*. *alvei*, but it has not been correlated with known OPS structures, and no previous report has described the genetic features of OPS. In this study, we obtained the genome sequences of 21 *H*. *alvei* strains (as defined by previous immunochemical studies) with different lipopolysaccharides. This is the first study to show that the O-antigen gene cluster in *H*. *alvei* is located between *mpo* and *gnd* in the chromosome. All 21 of the OPS gene clusters contain both the *wzx* gene and the *wzy* gene and display a large number of polymorphisms. We developed an O serotype-specific *wzy-*based suspension array to detect all 21 of the distinct OPS forms we identified in *H*. *alvei*. To the best of our knowledge, this is the first report to identify the genetic features of *H*. *alvei* antigenic variation and to develop a molecular technique to identify and classify different serotypes.

## Introduction

The genus *Hafnia* is one of over 40 genera that comprise the family *Enterobacteriaceae* [1]. Although Møller originally described this genus in 1954, the legitimacy of this group was constantly challenged for two decades, during which it was often referred to as “*Enterobacter alvei*” “*Enterobacter aerogenes* subsp. *hafniae*” or “*Enterobacter hafniae*” (www.bacterio.cict.fr/h/hafnia.html) [1, 2].

*Hafnia* spp. have been detected in food production units and recovered from the natural environment, including soil and water, but they have also been reported as opportunistic pathogens in hospital infections. *H*. *alvei* may cause acute gastroenteritis, and it is also regarded as an etiological factor in extra-intestinal diseases, primarily in immunocompromised patients [3, 4]. In 1996, Günthard and Pennekamp reported a large series of extra-intestinal *H*. *alvei* isolates and described their clinical significance [5].

Antigenic variation is one of the most important factors in pathogenicity and clonal adaptation, and it has been developed as the basis for defining clones within a number of species [6–10]. Lipopolysaccharide (LPS, endotoxin) is a major component of the cell wall in *H*. *alvei*. LPS consists of a lipid A anchor, a core oligosaccharide, and an O-polysaccharide chain (OPS, O-antigen) [6, 7, 11]. The OPS is the most variable portion of the LPS, and it dictates the serological specificity of Gram-negative bacteria [6, 7]. The OPS consists of oligosaccharide repeats (O-units) that normally contain two to eight sugar residues[10]. OPS variation lies predominantly in the types of sugars that are present in the molecule, their sequence in the structure, and the linkages that form between them [8, 9, 11, 12]. The presence of an OPS is essential for the survival of the bacteria in its natural environment, and the notion that OPS plays a role in bacterial virulence is supported by direct evidence showing that the loss of OPS makes many pathogens, such as *E*. *coli*, *Shigella*, *Francisella tularensis*, and *Yersinia enterocolitica*, serum-sensitive or otherwise seriously impairs their virulence [12, 13]. The genes that control OPS synthesis are normally present as a chromosomal gene cluster that maps between *galF* and *gnd* in *Salmonella*, *E*. *coli*, and *Shigella* [12]. However, one or more of these genes sometimes maps outside the gene cluster. These can include the genes in bacteriophages, which are often involved in modifying the structure of OPS and particularly in adding side-chain residues to the O-units. The OPS clusters consist of genes that belong to three main classes: nucleotide sugar biosynthesis pathway genes, glycosyltransferase genes, and O-unit processing genes [12, 13]. Three distinct pathways are involved in the synthesis and translocation of OPS: the Wzx/Wzy pathway, the ATP-binding cassette (ABC) transporter pathway, and the synthase pathway [12, 14]. In *E*. *coli* and *Shigella*, the Wzx/Wzy pathway is more often used. In this process, the O-unit is synthesized by the initial transfer of a sugar phosphate and the subsequently sequential transfer of other sugars from the respective sugar nucleotides to the carrier lipid, undecaprenyl phosphate (UndP). These O-units are flipped across the membrane while retaining their attachment to UndP, and they are then polymerized to form polysaccharide chains that are transferred to the independently synthesized core-lipid A to form lipopolysaccharide. In most *E*. *coli* and related bacteria, the initial transferase (IT) WecA transfers GlcNAc-1-P from UDP-GlcNAc to UndP and then 4-epimerase Gnu catalyzes conversion of UndPP-GlcNAc to UndPP-GalNAc [15, 16]. In *H*. *alvei*, however, the reported O-serotypes is not correlated with known OPS structures [12, 17–20], and no studies have examined the genetic features that characterize OPS variation, and none of the OPS gene cluster sequences were previously available.

In this study, we sequenced the genomes of 21 *H*. *alvei* strains that were defined by previous immunochemical studies and found that they had different LPS. The structures of 19 of these 21 strains have been published. This report is the first attempt to locate the OPS gene cluster in the *H*. *alvei* genome, which is located between *mpo* and *gnd* on the chromosome, and to reveal the genetic features of this gene cluster. We found that 15 OPS gene clusters out of the 19 strains for which OPS structures were available corresponded well with their OPS. In addition, the potential sugars that may be present in the OPS of the remaining two strains with no reported structures were summarized based on the OPS gene clusters sequenced by us. The presence of both the *wzx* and *wzy* genes in our sequenced strains suggested that OPS is produced via the Wzx/Wzy pathway in *H*. *alvei* [21]. The *wzx* and *wzy* genes in these 21 *H*. *alvei* strains were found to be polytropic, and the fact that each strain contained a unique *wzy* and *wzx* gene suggested a basis for rapid molecular detection. In this research, a PCR-based DNA suspension array that was based on the *wzy* genes was established to detect all 21 distinct OPS forms using one pair of specific primers plus one specific probe for each individual serotype. The microarray method described in this study is specific, sensitive, and reliable, and it may be a better alternative to the traditional serotyping procedure, which is laborious and frequently cross-reactive.

## Materials and Methods

### Strains conditions and genomic DNA extraction

*Hafnia alvei* strains were obtained from the Polish Collection of Microorganisms (PCM) at the Hirszfeld Institute of Immunology and Experimental Therapy, Polish Academy of Sciences (Wroclaw, Poland). All 21 *H*. *alvei* strains were used in this study, as shown in S1 Table. All of these strains were found to have different LPS using immunochemical methods. The *H*. *alvei* strains were grown in liquid medium as previously described [19] and then harvested using centrifugation (1380 x*g* for 15 min at 4°C). Genomic DNA was isolated using a Bacteria Extraction Kit (CWBIO Co., Ltd, China).

### Genome sequencing and analysis

Genome sequencing of PCM1220 was performed using Pac Bio RS II (Pacific Biosciences). The other 20 strains were sequenced using Solexa pair-end sequencing technology (Illumina, Little Chesterford, Essex).

For PCM1220, a 20-kb library was constructed and end-repaired, and the adaptors were then ligated to generate Single Molecule Real Time (SMRT) bells™ for circular consensus sequencing, with a depth of approximately 100-fold coverage. The sequencing data were *de novo* assembled using MaSuRCA [22, 23].

A Solexa Genome Analyzer IIx was used to sequence each isolate with a depth of 90- to 100-fold coverage. The Illumina data were *de novo* assembled using Velvet Optimiser v2.2 (http://bioinformatics.net.au/software.velvetoptimiser.shtml) [22]. Gaps within the gene clusters containing the major polysaccharide antigens were closed using PCR, and the products were then sequenced using ABI 3730 capillary sequencers (Applied Biosystems, U.S.A).

### Analysis of OPS gene clusters

TBLAST and PSI-BLAST [24] were used to search sequence databases, including the GenBank database and the Pfam protein motif database, to identify potential gene functions. The program TMHMM 2.0 [25] was used to identify potential transmembrane segments. Sequence alignments and comparisons were performed using the ClustalW program [26]. For sugar pathway genes, a BLAST search against the UniProt/SwissProt database was used to confirm the allocation of the genes by pathway [27].

The *wzx*, *wzy* and glycosyltransferase (GT) genes were individually allocated to homology groups (HGs) using the program OrthoMCL v2.0 [28] (http://orthomcl.org/common/downloads/software/v2.0/). A 50% amino-acid identity level was used as the cut-off. In the case of GTs, gene names were given directly, and for *wzx* and *wzy*, the serial numbers from *wzx*_1–21 or *wzy* 1-21were given (S2 Table).

The *wzx* and *wzy* phylogenetic trees were generated using both genes in all of the clusters. ClustalW v2.0 (http://www.ebi.ac.uk/Tools/msa/clustalw2/) was used to align the sequences, and phyML v3.0 (http://www.atgc-montpellier.fr/phyml/) and the JC69 module [29] were used to build a maximum likelihood tree for the 21 genomes.

### PCR amplification of the target *wzy* genes

PCR primers that corresponded to specific *wzy* genes were used to generate amplicons that were 80 to 600 bp long, depending on the OPS gene clusters (S3 Table) [30]. The forward primer was biotinylated at the 5’-end to allow it to bind the reporter dye and streptavidin–R-phycoerythrin, and it was subsequently detected using a Bio-Plex platform. The target sequences of all of the OPS gene clusters were amplified in a single multiplex PCR. PCR amplification was performed in 25-ul volumes using a Hot Start PCR kit (Promega, Madison, WI) according to the manufacturer’s instructions. PCR amplification was performed in a thermal cycler (MJ Research, MA) using the following parameters: an initial denaturation step at 95°C for 15 min; 30 cycles of 95°C for 30 s (denaturation), 50°C for 60 s (annealing), and 72°C for 90 s (extension); and a final extension step at 72°C for 10 min (final extension). PCR amplicons were then directly applied to the coupled beads in the hybridization reaction that is described below, and the O-group targets were detected in a single Bio-Plex assay.

### Probe design and bead coupling

All species-specific probes were designed based on the sequencing data obtained in this study. Multiple-sequence alignments were performed using BioEdit version 7.0 software. These species-specific probes were newly designed (S4 Table). The probes were synthesized with a 5’-end amino C-12 modification (AuGCT, Beijing, China) and coupled to carboxylated beads (Bio-Rad Laboratories, Hercules, CA) according to the instructions in the manufacturer’s manual. Briefly, 2.5 x 10^5^ carboxylated beads were suspended in 8.5 μL of 0.1 M MES (pH 4.5) with 2 μL of 0.1 nmol/μL oligonucleotide probes. A 2.5 μL volume of 10 mg/mL freshly prepared EDC was added, and the mixture was immediately vortexed. It was then incubated at room temperature in the dark for 30 min. This step was repeated a second time. After the beads were washed with 0.02% Tween 20 and 0.1% SDS, the pellets were centrifuged and resuspend in 20 μL of TE (pH 8.0) and then stored at 4°C in the dark until used.

### Hybridization and staining

A bead mix set was prepared for each of the 10 probes. The mix consisted of 2,500 beads in a 1.5x tetramethylammonium chloride (TMAC) solution (Sigma, St. Louis, MO) containing 4.5 M TMAC, 0.15% Sarkosyl, 75 mM Tris-HCl (pH 8.0), and 6 mM EDTA (pH 8.0). A total of 17 μL of the biotinylated amplicon was added to 33 μl of the bead mix. The amplicon and bead mixture was then denatured at 95°C for 5 min and allowed to hybridize at 55°C for 15 min. The mixture was centrifuged at 8,000 rpm, and the supernatant was then carefully discarded. The beads were resuspended in 75 μL of 1x TMAC solution containing 10 ng/mL streptavidin-R-phycoerythrin (Molecular Probes, Eugene, OR) and incubated for 10 min at 55°C.

### Suspension array data acquisition and analysis

The beads were analyzed based on fluorescence intensity using a Bio-Plex 100 suspension array system (Bio-Rad Laboratories). The median fluorescence intensities (MFI) were calculated from 100 replicate measurements that were obtained using a digital signal processor and BioPlex Manager 4.1 software. A positive signal was defined as a MFI of at least >500 and a signal/background ratio (S/B ratio = MFI/Blank) that was greater than six.

## Results and Discussion

### Identification and location of OPS clusters

**Fig 1:**
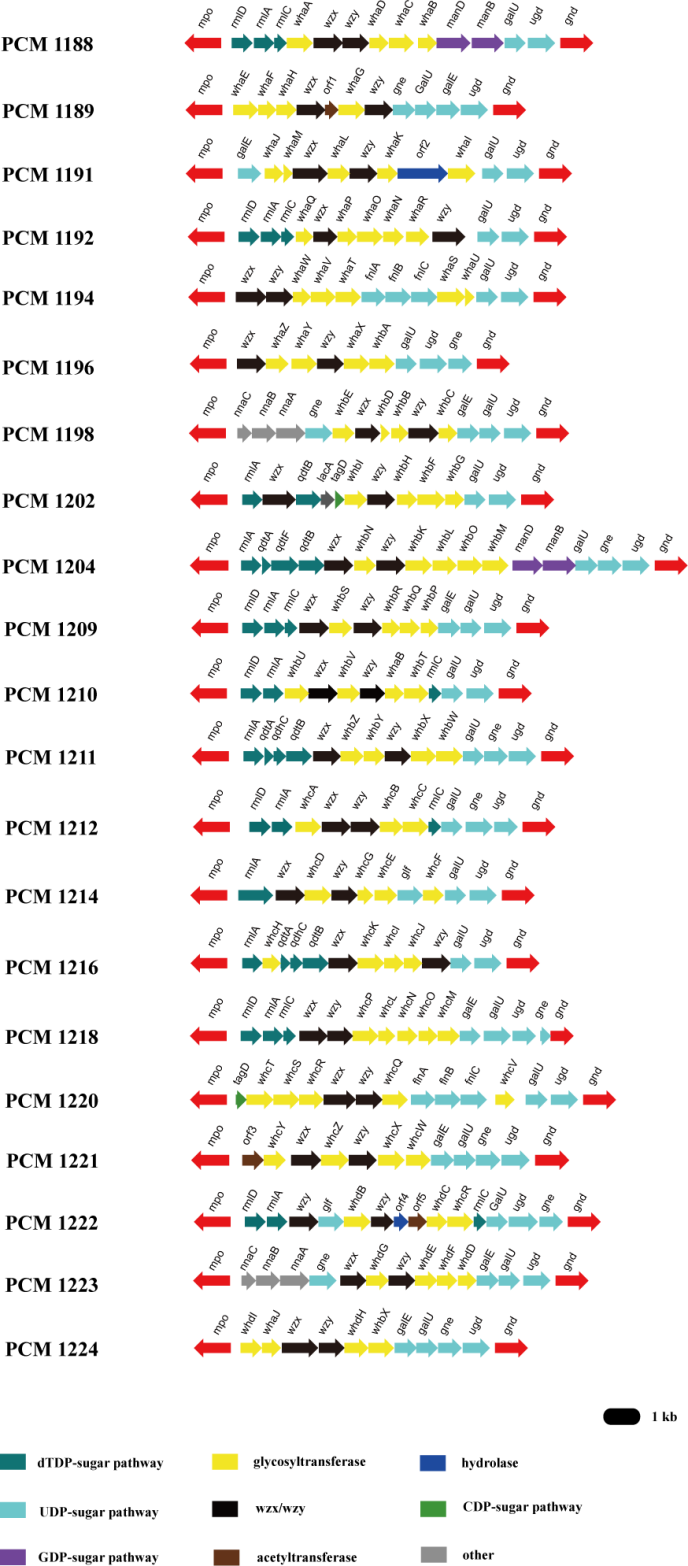
The polysaccharide gene clusters in the 21 *H*. *alvei* type strains. The sequences of 21 *H*. *alvei* OPS gene clusters have been deposited in Genbank, with the accession numbers from KX117077 to KX117097.

**Fig 2:**
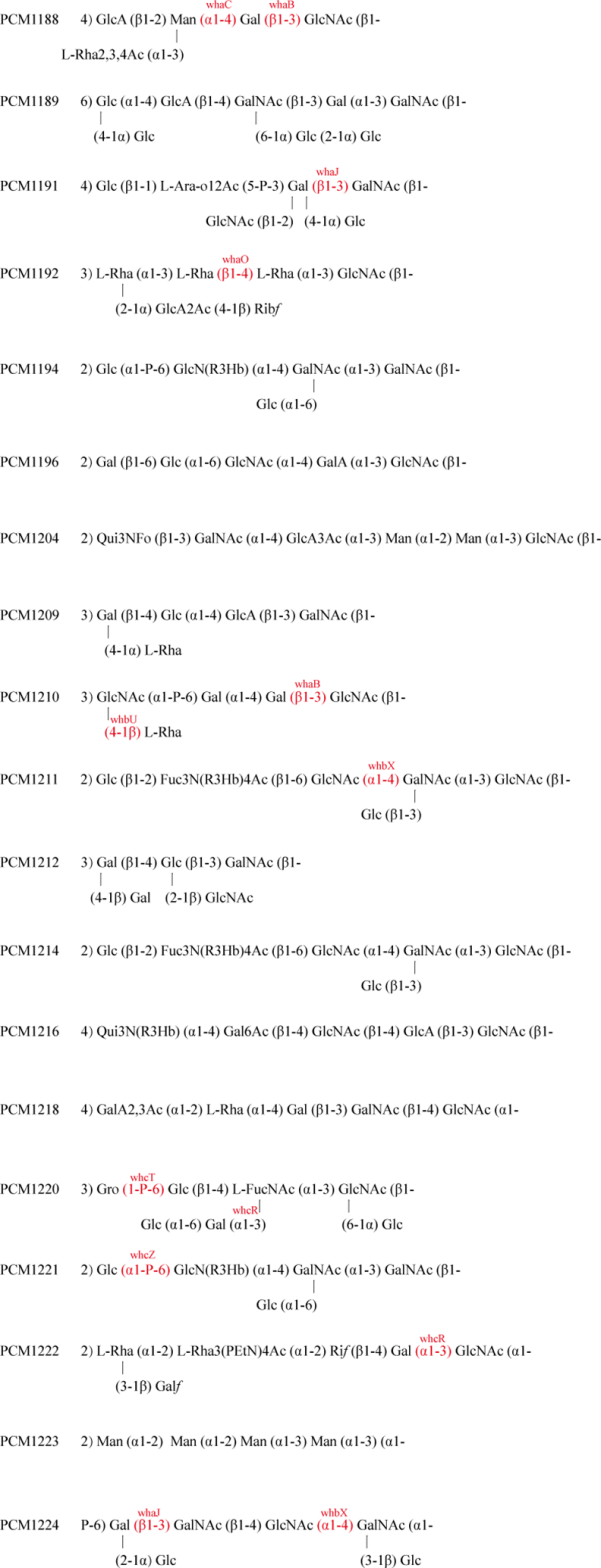
The available structures for the type strains. The gene names shown against some of the glycosidic linkages are the GT genes that are proposed to be responsible for that linkage. For abbreviations, see Fig 3 legends.

**Fig 3:**
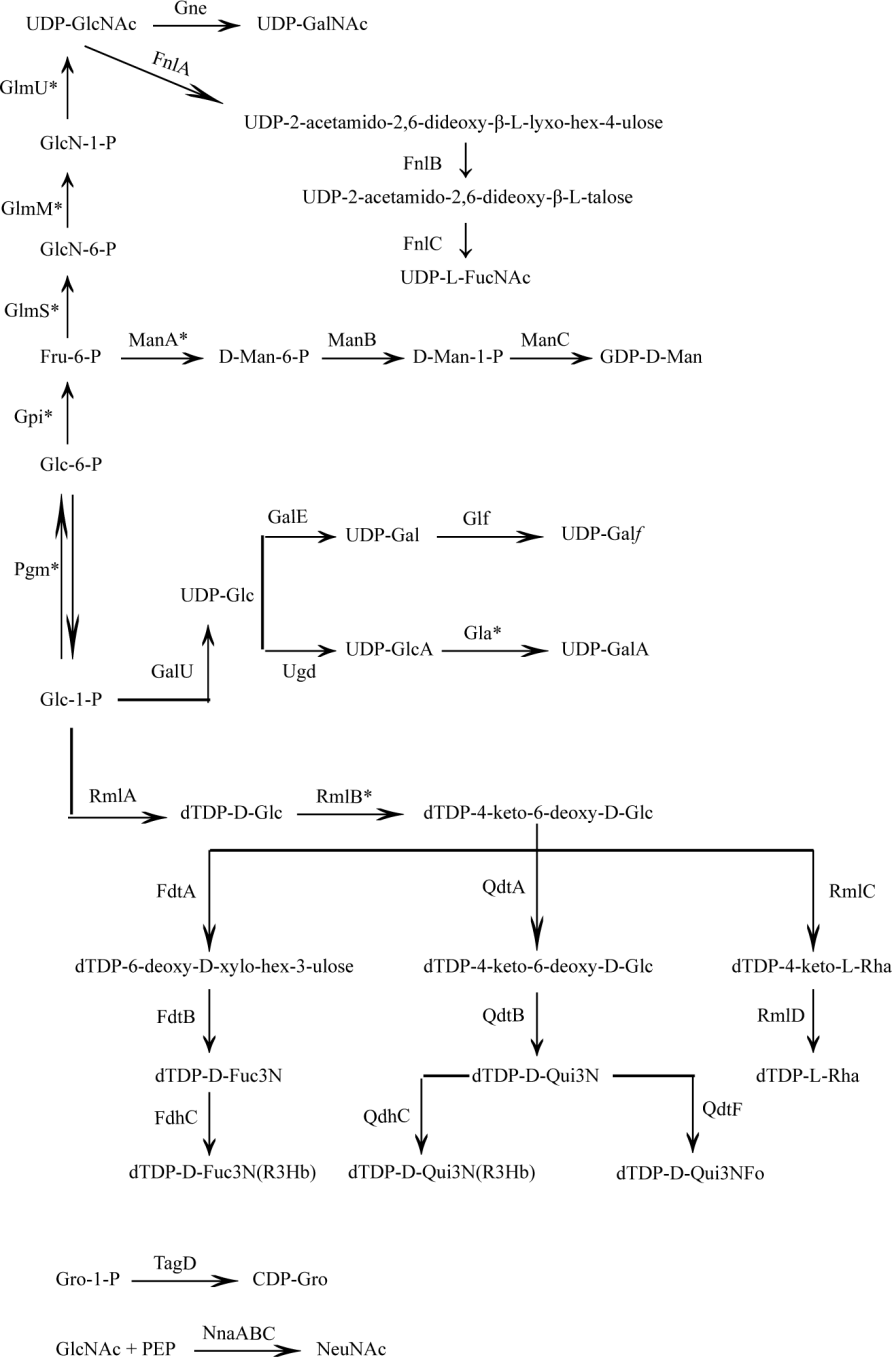
Proposed biosynthesis pathways for sugars in *H*. *alvei* major polysaccharides. The abbreviations in the structures: Ac, O-acetyl; Ara-ol, arabinitol; Glc, D-glucose; GlcA, D-glucuronic acid; GlcN, 2-amino-2-deoxy-D-glucose; GlcNAc, 2-acetamido-2-deoxy-D-glucose; Gal, D-galactose; GalA, D-galacturonic acid; Gal*f*, D-galactofuranose; GalNAc, 2-acetamido-2-deoxy-D-galactose; Gro, Glycerol; Gro-1-P, Glycerol-1-P; L-Rha, L-rhamnose(6-deoxy-L-mannose); Fo, formyl; Fru, beta-D-fructose; L-FucNAc, 2-acetamido-2-deoxy-L-fucose; D-Fuc3N, 3-amino-3-deoxy-D-fucose; D-Fuc3N(R3Hb), 3-[(R)-3-hydroxybutanoylamino]-3-deoxy-D-fucose; PEtN, phosphoethanolamine; R3Hb, (R)-3-hydroxybutanoylamino; Ri*f*, ribofuranose; D-Man, D-mannose; D-Qui3N, 3-amino-3-deoxy-D-quinovose; and D-Qui3N(R3Hb), 3-[(R)-3-hydroxybutanoylamino]-3-deoxy-D-quinovose; * indicates the genes that were found outside the OPS cluster.

We obtained the whole genome sequence of the *H*. *alvei* strain PCM1220. A potential OPS gene cluster was predicted that consisted of nucleotide sugar biosynthesis pathway genes, glycosyltransferase genes, and the O-unit processing genes *wzx* and *wzy*. This potential OPS cluster was located between the *mpo* (*membrane protein outside-cluster)* and *gnd* genes in the chromosome of PCM1220 (Fig 1). We then compared this potential OPS gene cluster to the reported PCM1220 OPS structure to check for correspondence. The OPS of PCM1220 contains 5 types of sugar residues: Gal, Glc, GlcNAc, FucNAc and Gro (Fig 2). Among these, Glc,Gal and GlcNAc are thought to be common sugars, which are involved in basic cell metabolism and are synthesized by well-known genes through processes that were covered in a recent review [12]. There is also a *galU* gene, which is required to synthesize UDP-Glc, in the OPS gene cluster of PCM1220 (Figs 2 and 3). It should be noted that this gene was found in all 21 *H*. *alvei* OPS gene clusters included in this study (Fig 1). FnlABC, which is responsible for the synthesis of UDP-L-FucNAc, has been reported to be involved in synthesizing O-polysaccharides in several species, including *E*. *coli* O117 [12]. The set of *fnlABC* genes in PCM1220 shares more than 77% identity with the corresponding genes in *E*. *coli* O145. There is a glycerol residue in the structure, and a *tagD* was found in the cluster that shares 89% identity with the *E*. *coli* gene. The product of this gene acts similar to glycerol-3-phosphate cytidylyltransferase, which converts Glycerol-1-P to CMP-Gro [31]. There are two Glc residues in the side chain of the OPS structure in PCM1220. Normally, the *gtr* operon outside the OPS gene cluster, which consists of *gtrA*, *gtrB* and several GT genes that are aligned in the same direction, is thought to be responsible for the side-chain glycosidic linkages in OPS [32]. Briefly, the side-branch sugar residues are added during a three-step process that involves GtrA and GtrB, which are common to all such residues, and a side-branch-specific transferase. This process has been described in a recent review [12]. As expected, we found a *gtr* operon in the chromosome outside of the OPS cluster in PCM1220 (S1 Fig), which is proposed to be responsible for one of the side-chain Glc-related linkages. In addition, we identified five transferase genes (Fig 1 and S5 Table) in the OPS gene cluster, which are proposed to be responsible for the remaining four glycosidic linkages and one glycerol 1-phosphate linkage, as expected. Thus, there is a good co-relation between genes and structures in PCM1220. These data demonstrate that the content of the OPS gene cluster between the *mpo* and *gnd* genes in the chromosome correlates with the OPS structure in PCM1220. This is the first attempt to reveal the location of the OPS gene cluster in *H*. *alvei*, in which it is located between *mpo* and *gnd* in the chromosome.

### Analysis of the OPS clusters in another 20 sequenced *H*. *alvei* strains

We obtained the draft genome sequences of additional twenty strains of *H*. *alvei* (S1 Table). Thus, a total of 21 OPS gene clusters, including that of PCM1220, were extracted in this study, and all of them were located between the *mpo* and *gnd* genes (Fig 1). The clusters that were extracted from these 21 strains were highly diverse, with each different strain sequence representing one of the 21 serotypes.

Each OPS gene cluster included a set of *wzx* and *wzy* genes, which suggested the presence of Wzx/Wzy pathway-related OPS processing. We then compared these OPS gene clusters to reported OPS structures. As expected, we found a high level of correlation among 15 of the sequenced *H*. *alvei* strains, including PCM1220, PCM1188, PCM1189, PCM1191, PCM1192, PCM1194, PCM1196, PCM1209, PCM1210, PCM1211, PCM1216, PCM1218, PCM1221, PCM1222 and PCM1224 (Figs 1 and 2), but not for the following four strains: PCM1194, PCM1212, PCM1214 and PCM1223. The details of these findings are discussed below.

#### PCM1188

The *manABC* genes are required to synthesize GDP-D-Man [10]. We found *manBC* within the cluster and *manA* outside the cluster, and we showed that they share 89% and 83% identity, respectively, with the corresponding genes in *E*. *coli* [12]. A rare sugar, L-Rha2,3,4Ac, was found in the OPS structure of PCM1188. Rha is widely present in bacterial surface polysaccharides, and its biosynthesis pathway is well known to involve four enzymes (RmlABCD) that are encoded by genes in the polysaccharide gene clusters of *E*. *coli*, *Shigella*, *Acinetobacter* and *Salmonella* [12]. For PCM1188, the genes *rmlCAD*, which showed more than 80% identity to the corresponding homologs in *E*. *coli* O189 [10, 12], were found in the OPS gene clusters, and *rmlB* was found outside the cluster but within the chromosome, near to the IT gene *wecA*, which we will discuss below. Furthermore, we found that *rmlABCD* genes were present in all *H*. *alvei* strains that were examined in this study that contained a Rha residue in the OPS (Fig 1). Because the O-acetyltransferase genes that are required to synthesize L-Rha2,3,4Ac from L-Rha were not found in the cluster, we inferred that the related genes may be present in the chromosome but outside the OPS gene cluster, as has been reported in other species. In addition, there were four glycosidic linkages in the structure, and we found four GT genes in the gene cluster, as expected.

#### PCM1189

Gne functions during the conversion of UDP-GlcNAc to UDP-GalNAc. In the structures described in this paper, it is common to find a *gne* gene in clusters that contain GalNAc. There are seven glycosidic linkages in the OPS of PCM1189, and three of them are on side chains. The four GT genes in the cluster are proposed to be responsible for the four linkages in the main chain. A 369-bp Orf in PCM1189 that had 98% query coverage and 56% identity to GtrA in the *E*. *coli* chromosomewas annotated to GtrA, which has been discussed above (S1 Fig). However, the GtrA in PCM1189 is on an 800-bp assembled segment, and we found only a 51-bp ORF residue, which was terminated by the gap, in the region downstream of GtrA. These data infer that there is a *gtr* operon in the PCM1189 chromosome that controls side-chain glycosidic linkages. Therefore, we inferred that the side-chain glycosidic linkages in this structure are transferred by genes that are located outside of the OPS gene cluster. In addition, there is an extra putative O-acetyl transferase gene in the gene cluster, but the OPS contains no related acetyl. Apart from these data, we found a good correlation between genes and structures.

#### PCM1191

The *galE* is the gene that is responsible for the synthesis of UDP-Gal in the gene cluster. This information has been extensively covered in recent reviews [12] and will not be discussed here. We also found an Ara-ol2Ac in PCM1191, which is rarely found in bacterial surface polysaccharides. The genes that synthesize this sugar residue were not present in the OPS gene cluster, and we therefore proposed that these genes may be located outside the cluster. In addition, there were four glycosidic linkages and one glycerol 1-phosphate linkage, and we found five transferase genes in the cluster, showing a good correlation.

#### PCM1204

There is a Man in the OPS structure of PCM1204, and a set of *man* genes, which are required to synthesize GDP-D-Man, were identified in the OPS gene cluster. Another rare sugar is Qui3NFo. As reported in *E*. *coli* O114, RmlA and QdtAB convert Glc-1-P to dTDP-D-Qui3N, and QdtF converts Qui3N to Qui3Nfo [10]. The *rmlA* and *qdtAB* genes in PCM1204 share more than 70% identity with the respective genes in *E*. *coli* O114 [33]. In addition, the five GT genes are proposed to be responsible for five glycosidic linkages.

#### PCM1211

Fuc3N (R3Hb) was identified in the OPS structure of PCM1211. As was previously reported in *E*. *coli* O103, RmlA and FdtAB convert Glc-1-P to dTDP-D-Fui3N, and FdhC converts Fui3N to Fuc3N(R3Hb) [34]. The *rmlA*, *fdtAB* and 3-hydroxybutanoyl transferase gene *fdhC* were also found in PCM1211, and these genes each share more than 68% identity with the corresponding genes in *E*. *coli* O103 [35]. There is a side-chain glycosidic linkage in the OPS of PCM1211 that indicates the presence of a *gtr* operon, which adds side branch glucose residues, in the chromosome. As expected, *gtrA* was identified in the assembled segments of the chromosome. The other four GT genes are proposed to be responsible for the remaining four glycosidic linkages in the main chain.

#### PCM1216

A rare sugar, dTDP-D-Qui3N (R3Hb), was identified in the OPS structure of PCM1216. A set of *qdtAB* genes and *rmlA*, which is required to synthesize dTDP-Qui3N [33] and was discussed in the PCM1204 section, were identified in PCM1216. As reported in *Acinetobacter* Sv23, the 3-hydroxybutanoyl transferase QdhC is required to synthesize dTDP-D-Qui3N(R3Hb) from dTDP-Qui3N[35], and we found that the *qdhc* in PCM1216 shares more than 52% identity with the corresponding gene in *Acinetobacter* Sv23. There are four GT genes, and these genes are proposed to be responsible for the four glycosidic linkages.

#### PCM1221

Because *orf3* shares approximately 35% identity with a putative transferase in *E*. *coli* O103, we inferred that it might act as a 3-hydroxybutanoyl transferase, which is required to synthesize GlcN(R3Hb) from GlcN. There are four GT genes, and we found four glycosidic linkages, as expected.

#### PCM1222

A rare sugar, L-Rha3(PEtN)4Ac, was found in the structure of PCM1222. In PCM1222, two putative sugar biosynthesis pathway genes, *orf04* and *orf05*, share approximately 32% and 30% identity with a haloacid dehalogenase-like hydrolase and a putative acyltransferase, respectively, in *E*. *coli* MS 119–7. Because there is an acetyl-related structure in Rha3(PEtN)4Ac, we propose that one or both of these two genes may be required to synthesize L-Rha3(PEtN)4Ac from L-Rha. The function of *glf* is to convert UDP-Gal to UDP-Gal*f*. The gene that is required to synthesize Rib*f* may be present in the chromosome but outside the cluster, similar to what has been observed in *E*. *coli* [17]. There are three remaining glycosidic linkages, but there are only two GT genes in the cluster. We found a *gtrABC* operon in the chromosome, suggesting the presence of side branch residues, as discussed for PCM1189. Thus, there is a good co-relation between genes and structures.

#### PCM1192 / PCM1196 / PCM1209 / PCM1210 / PCM1218 / PCM1224

We found a set of *rml* genes, which are required to synthesize dTDP-L-Rha, in PCM1192, PCM1209, PCM1210 and PCM1218. All of these contain Rha residues. We found a *gtr* operon, which is required to add a side branch of OPS glucose residues, in the PCM1224 chromosome. Moreover, we found a good correlation between GT genes and glycosidic linkages in the OPS structures of PCM1192, PCM1196, PCM1209, PCM1210, PCM1218 and PCM1224.

#### PCM1194 / PCM1212 / PCM1214 / PCM1223

Unlike the above 14 well structure-matched clusters, gene clusters in PCM1194 and PCM1223 could not be strongly linked to their reported structures [36]. A set of *fnl* genes, which are required to synthesize L-FucNAc [12, 37], was identified in the PCM1194 cluster, but there was no L-FucNAc in its structure. We identified a GlcN(R3Hb) in its structure, but no related genes that could be involved in the synthesis of R3Hb or transferase were identified in the cluster. A set of *nna* genes, which are required to synthesize NeuNAc [38], were identified in the gene cluster of PCM1223, but no NeuNAc was identified in its structure. There were also no Man-related genes in its cluster. A set of *rml* genes, which is required to synthesize L-Rha, was identified in the PCM1212 and PCM1214 cluster, but there was no L-Rha in both structures. And there is a *glf* gene in the cluster of PCM1214, but no Gal*f* present in the PCM1214 structure. In these four cases, there may have been errors during strain maintenance or transfer between labs. Hence, the names PCM1194, PCM1212, PCM1214 and PCM1223 are being maintained for these four strains.

No structures were available for the remaining two strains, PCM1198 and PCM1202. Based on their OPS clusters, we predict that there is a set of *nna* genes in the PCM1198 cluster [19] and that NeuNAc may be present in PCM1198 [38]. There is a *rmlA* gene and a *qdtB* gene in the PCM1202 cluster [35], indicating that Qui3N may be present in PCM1202 [35].

In summary, among the 21 strains for which different lipopolysaccharides were defined in previous immunochemical studies, a total of 15 sequenced gene clusters in *H*. *alvei* strains were found to show a good correlation between the genes that were present and their associated structures, with the exceptions of two gene clusters for which OPS structural data was not available and four gene clusters that were unrelated to their structures. In each OPS gene cluster, there are always 8–14 genes, which are generally related to OPS specificity, and they are followed by a set of genes (*galU*, *gne* and *gnd*) at the 3’ end of the gene cluster that is not, in general, related to serotype specificity. All of the genes except the *mpo* gene are transcribed in the same direction (Fig 1). The *galU*, *ugd*, *gnd* and *mpo* genes are present in all of the analyzed OPS clusters. Interestingly, in *H*. *alvei*, there are two *galU* genes in the chromosome: one in the OPS cluster and another nearby in the IT *wecA*. The gene *galU* is usually located outside the OPS cluster in *Salmonella* and *E*. *coli*, but it has been reported to be present within the OPS cluster in *Acinetobacter*. Hence, the presence of two *galU* genes and an extra cluster for side-chain glycosidic linkages in the chromosome suggests that these genes were horizontally transferred at different times during evolution. In addition, the GC percentage in these OPS clusters as around 38%, which is significantly lower than the GC percentage in the whole genome (50%-52%). This suggests that the OPS gene cluster in *H*. *alvei* may have been transferred from species with lower GC percentages. Each set of strain-specific genes includes both a *wzx* and a *wzy*, and this indicates that the OPS in *H*. *alvei* are Wzx/Wzy pathway-dependent.

### The allocation of IT and GTs to special linkages

In most *E*. *coli* and *Shigella* strains, the first sugar residue in the O-unit is GlcNAc or GalNAc, and the IT encoded by *wecA* is responsible for initiating the synthesis of GlcNAc- and GalNAc-initiated OPS. Usually, the WecA gene is located outside the cluster and acts as a UDP-GlcNAc:undecaprenylphosphate GlcNAc-1-phosphate transferase. Genes that encode ITs are usually conserved across different species, and we found support for the homology of the *wecA* gene across all 21 *H*. *alvei* strains, which share more than 70% identity with the corresponding gene in *E*. *coli* and *Shigella* and more than 90% identity with the corresponding gene in *Yersinia*. In 18 of the 19 *H*. *alvei* OPS structures discussed in this study, we found GalNAc or GlcNAc, and we propose that in most *H*. *alvei* strains, *wecA* transfers the UDP-GlcNAc to the undecaprenylphosphate as the first sugar of the repeated unit, while the Gnu is responsible for the conversion of UndPP-GlcNAc to UndPP-GalNAc [16]. The only exception was the OPS of PCM1223, which contains only Man, but not GalNAc or GlcNAc. However, the gene cluster in PCM1223 could not be linked to this reported structure, as discussed above.

Glycosyl transferases sequentially add sugars to growing sugar chains until the O-unit/OPS has been fully synthesized. The extensive variety of sugars that have been found in OPS allows the formation of numerous combinations of donor sugar, acceptor sugar and acceptor carbon atom in glycosidic linkages. This variety also supports a very large number of linkage specificities and therefore glycosyl transferase specificities. In this study, a total of 54 different glycosidic linkages were available in the structures of the strains we sequenced, suggesting the presence of a high degree of diversity in GTs (S5 Table). The 85 putative GT genes that were identified in the 21 discrete sequences were allocated to 50 homology groups and named HG01-HG50, as shown in S5 Table. We were able to provisionally allocate nine of these, including *whaB*, *whaJ*, *whbX*, *whcR*, *whaO*, *whcT*, *whaC*, *whbU* and *whcZ*, to specific functions based on homologies that were determined using BLAST searches. These are listed below and the presence of linkages that were shared by different polysaccharide structures is discussed in Fig 2.

Generally, GTs belonging to the same homology group (HG) are thought to perform the same or highly similar functions. For instance, there are nine GTs in the HGs of HG08, and they share 35–95% identity in pairwise comparisons. Among these, *whaB* is present in the clusters of PCM1188 and PCM1210, and there is accordingly only one common Gal-(β1–3)-GlcNAc linkage in these two OPS structures. Hence, *whaB* was proposed to have a function that is putatively responsible for a Gal-(β1–3)-GlcNAc linkage. After further considering the structural data, including the Gal-(β1–3)-GalNAc linkages in the OPS structures of PCM1191 and PCM1224 and the fact that there is a *whaJ* that belongs to HG08 in both the PCM1191 and the PCM1224 cluster, *whaJ* was proposed to play a role in the formation of the Gal-(β1–3)-GalNAc linkage in the both of these OPS structures. Similarly, a single and identical GlcNAc-(α1–4)-GalNAc linkage was identified in PCM1211 and PCM1224, and the presence of *whbX* in the OPS gene cluster in each of these strains suggests that *whbX* is responsible for the GlcNAc-(α1–4)-GalNAc linkage. Because we found a single, identical Gal-(α1–3)-GlcNAc linkage in both the PCM1220 and the PCM1222 structure, the *whcR* in these two strains is proposed to be functionally responsible for the Gal-(α1–3)-GlcNAc linkage.

In addition to the above four GTs, for which we have proposed functions based on a classified homology analysis, five additional GTs were also allocated by our functional predictions, and these shared more than 50% identity with known GT genes, such as *whaC* in PCM1188, which shares 64% identity with a mannosyl transferase in *E*. *coli* and is therefore proposed to be required for Man-(α1–4)-Gal linkages. Similarly, the putative glycerol phosphate transferase *whcT* is proposed to function in Gro-(1-P-6)-Glc linkages in PCM1220, the putative rhamnose transferase *whaO* is proposed to function in L-Rha-(β1–4)-L-Rha linkages in PCM1192, the putative rhamnose transferase *whbU* is proposed to function in L-Rha-(β1–4)-GlcNAc linkages in PCM1210, and the putative glucose phosphate transferase *whcZ* is proposed to function in Glc-(α1-P-6)-GlcN(R3Hb) linkages in PCM1221.

### Homology groups for Wzy/Wzx

**Fig 4:**
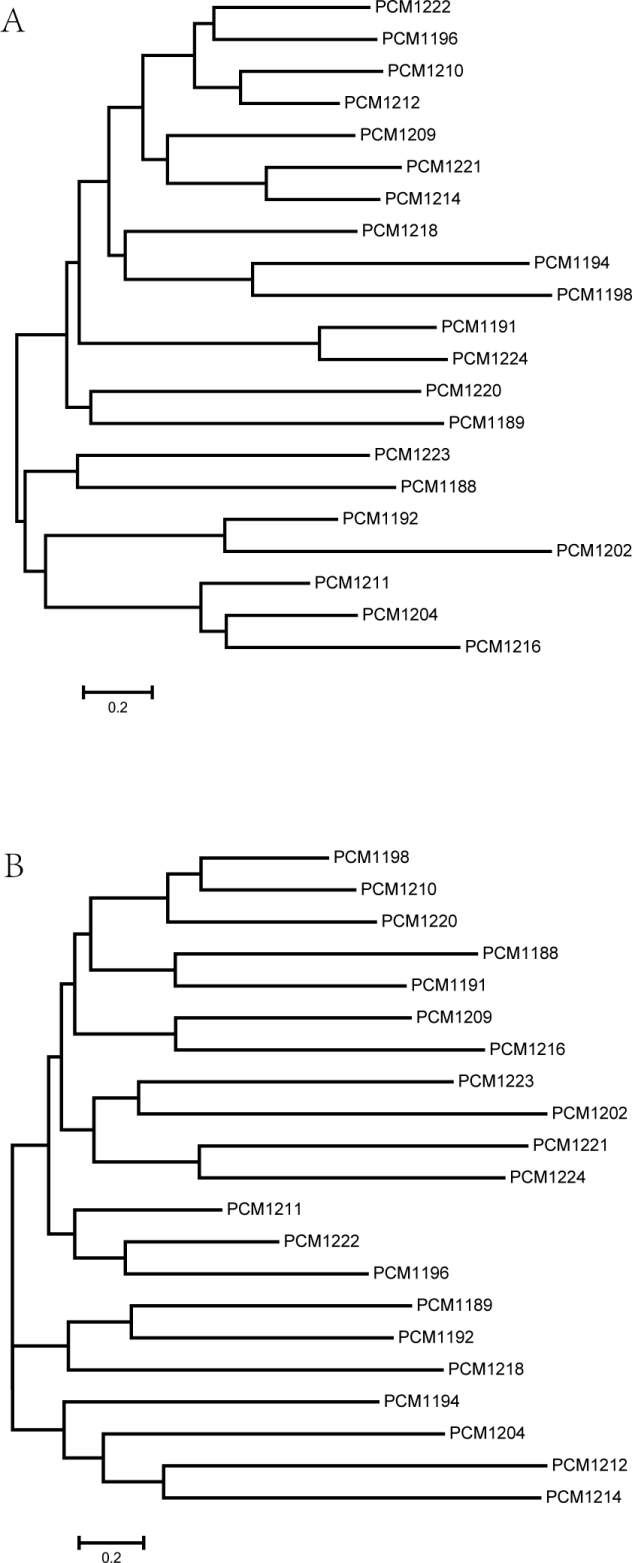
The phylogenetic trees for *wzx* and *wzy* in the *H*. *alvei* strains. The (A) *wzx* and (B) *wzy* trees were constructed using these two genes, which were found in all clusters, respectively. The sequences were aligned using ClustalW v2.0. The trees were generated using phyML v3.0 and the JC69 substitution model.

We identified distinctive forms of both Wzy and Wzx using unique serial numbers that were based on HGs in orthoMCL (see Materials and Methods). Each of the 21 different clusters has a unique Wzy HG and a unique Wzx HG (S2 Table). All of the *wzx* genes encode proteins with 10 to12 transmembrane segments, as expected, and all of the *wzy* genes encode proteins with 9 to 12 transmembrane segments. We generated phylogenetic trees using these Wzx HGs and Wzy HGs (Fig 4). The diversity of *wzx* and *wzy* genes provided us with the opportunity to apply molecular techniques to identify and classify different serotypes with the aim of developing a process that can be used to diagnose *H*. *alvei* infections.

### PCR-based suspension arrays for molecular serotyping of 21 serogroups

Because traditional antiserum serotyping methods are limited, PCR-based molecular serotyping was developed using O antigen-specific genes for several different serogroups of species, including *E*. *coli* and *Salmonella* [12]. According to the results of the protein clustering analysis that was performed in this study, the non-initial GTs Wzx and Wzy were specific to distinct OPS types, indicating that these genes could be selected for genotyping. In this study, we established the approach of using the Wzy as an ideal target gene for serotype detection.

Primers were designed to obtain *wzy* PCR amplicons, as described in the materials and methods. We targeted *wzy* to generate amplicons of each serotype strain. Initially, all of the forward primers were used at 40 nM, while the reverse primers were used at 160 nM. However, several strains failed to generate the expected hybridization signals under these conditions. Consequently, the primer concentrations were adjusted to 80 nM for the forward primers and 320 nM for the reverse primers. Under these more optimized conditions, the *wzy*-PCR amplicons of all 21 of the *H*. *alvei* strains were amplified, and the lengths of their PCR products varied from 91 to 249 bp. Then, capture probes that were 18 to 22 bp in length were designed for each serotype. Probe hybridizations were evaluated at different temperatures (37°C, 52°C, and 55°C), and the fluorescence signal intensity and stringency of hybridization were found to be optimal at a hybridization temperature of 55°C. For all 21 of the tested strains, the S/B ratio for each probe that was tested against its homologous DNA was significantly greater than the ratio that was obtained when the probes were tested against nonhomologous DNA, with the S/B ratios of the positive samples ranging from 2.0 to 5.0. No cross reactivity was observed for any probe that was tested against nonhomologous DNA (S2 Fig).

### The sensitivity and reproducibility of a suspension array using genomic DNA

To assess the sensitivity of the suspension array, a ten-fold dilution series (including 0 fg/μL, 1.0 fg/μL, 10.0 fg/μL, 100.0 fg/μL, 1.0 pg/μL, 10.0 pg/μL, 100.0 pg/μL, 1.0 ng/μL, 10.0 ng/μL and 100.0 ng/μL) of genomic DNA derived from the target bacteria was used as the template for multiplex PCR. Positive signals were generated for templates that contained 10–100 pg of genomic DNA. To test the reproducibility of the bead-based suspension assay, the PCR and hybridization reactions were performed in three parallel runs using 100 ng of genomic DNA from each target species. The interassay variation (CV %) that was obtained from this test ranged from 4.24%–10.85%.

Taken together, these data show that the probes and primers we designed worked well on the target strains and resulted in no non-specific signals. This suspension array, like other molecular detection assays, has its limitations. Any probe that is used in such an assay must be designed based on a known sequence. However, once a new OPS is identified using sequencing, probes can be quickly and easily designed based on the OPS sequence to complement our microarray. Overall, our *wzy*-based suspension model provides a potential tool that can be used to identify the OPS of a given *H*. *alvei* strain.

## Conclusions

OPS are important components of the outer membranes of Gram-negative bacteria and highly variable with substantial variation within and between different species. In this study, we sequenced the genomes of 21 *H*. *alvei* strains that were found to have different lipopolysaccharides in previous immunochemical studies. We identified 21 OPS gene clusters for the first time that were located between *mpo* and *gnd* on the strain chromosomes in *H*. *alvei*. Among the 19 strains with available OPS structures, we found that 15 of the gene clusters correlated well with their reported structures. The presence of both the *wzx* and the *wzy* gene in our sequenced strains suggests that OPS in *H*. *alvei* are processed by the Wzx/Wzy pathway and that they are highly diversified, and the variation in these genes suggests that OPS may be a useful resource for rapidly detecting clinical pathogenic isolates. We therefore designed specific primers and probes for a suspension array that showed can distinguished each of these 21 serotypes, thereby providing an easy-to-use, high-throughput tool for rapidly detecting *H*. *alvei* in clinical assessments and for facilitating better control of the disease.

## Supporting Information

S1 Fig The available *gtr* operon for the respective OPS synthesis. (DOCX)

S2 Fig The hybridization results of 21 *H*. *alvei* strains. (DOCX)

S1 Table The strains and the accession numbers in this study. (DOCX)

S2 Table The *wzx* and *wzy* forms. (DOCX)

S3 Table The primers used in this study. (DOCX)

S4 Table The probe used in this study. (DOCX)

S5 Table The GT names and HGs. (DOCX)
