# Supplementary figures and images for: Genetic Diversity of O-Antigens in Hafnia alvei and the Development of a Suspension Array for Serotype Detection
Source: PLoS One. 2016 May 12;11(5):e0155115. doi: 10.1371/journal.pone.0155115 (PMC4869667; doi:10.1371/journal.pone.0155115)

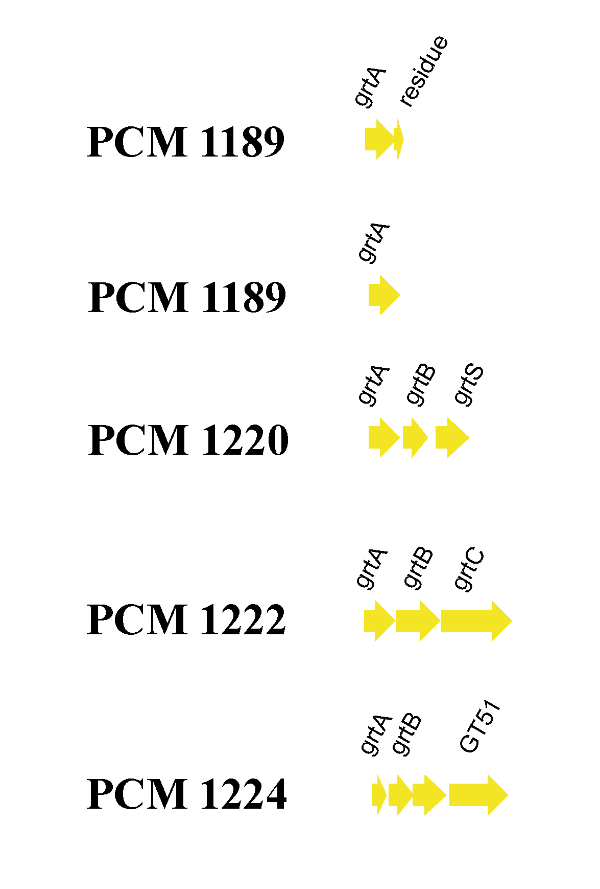


**Fig S1. The available *gtr* operon for the respective OPS synthesis.**

Supplement: S1 Fig — (DOCX) [file pone.0155115.s001.docx]
