# Supplementary material for: Genetic Diversity of O-Antigens in Hafnia alvei and the Development of a Suspension Array for Serotype Detection
Source: PLoS One. 2016 May 12;11(5):e0155115. doi: 10.1371/journal.pone.0155115 (PMC4869667; doi:10.1371/journal.pone.0155115)
Supplement: S2 Fig — (DOCX) [file pone.0155115.s002.docx]

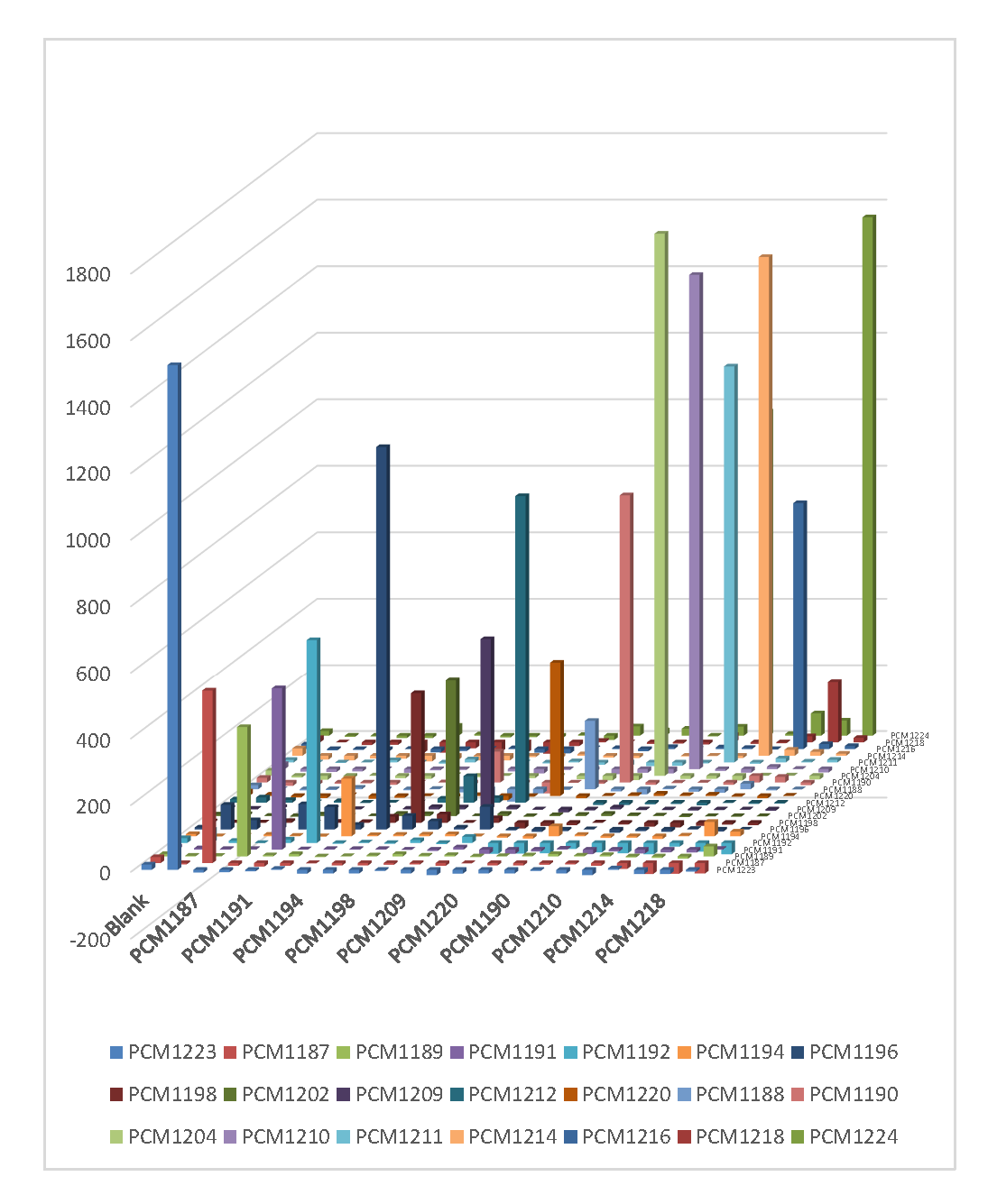


**Fig S2. The hybridization results of 21 *H. alvei* strains.** The specific probe of each of 21 strains was tested against nonhomologous DNA and the Blank was a negative control.
