# Supplementary material for: Genetic Diversity of O-Antigens in Hafnia alvei and the Development of a Suspension Array for Serotype Detection
Source: PLoS One. 2016 May 12;11(5):e0155115. doi: 10.1371/journal.pone.0155115 (PMC4869667; doi:10.1371/journal.pone.0155115)
Supplement: S1 Table — (DOCX) [file pone.0155115.s003.docx]

**Table S1. The strains used in this study**

| **Strains** | **Lab. Collection Number** | **Species** | **Accession Number** |
| --- | --- | --- | --- |
| PCM 1188 | G5899 | *H. alvei* | KX117077 |
| PCM 1189 | G5900 | *H. alvei* | KX117078 |
| PCM 1191 | G5902 | *H. alvei* | KX117079 |
| PCM 1192 | G5903 | *H. alvei* | KX117080 |
| PCM 1194 | G5904 | *H. alvei* | KX117081 |
| PCM 1196 | G5906 | *H. alvei* | KX117082 |
| PCM 1198 | G5907 | *H. alvei* | KX117083 |
| PCM 1202 | G5908 | *H. alvei* | KX117084 |
| PCM 1204 | G5910 | *H. alvei* | KX117085 |
| PCM 1209 | G5913 | *H. alvei* | KX117086 |
| PCM 1210 | G5914 | *H. alvei* | KX117087 |
| PCM 1211 | G5915 | *H. alvei* | KX117088 |
| PCM 1212 | G5916 | *H. alvei* | KX117089 |
| PCM 1214 | G5918 | *H. alvei* | KX117090 |
| PCM 1216 | G5920 | *H. alvei* | KX117091 |
| PCM 1218 | G5922 | *H. alvei* | KX117092 |
| PCM 1220 | G5895 | *H. alvei* | KX117093 |
| PCM 1221 | G5898 | *H. alvei* | KX117094 |
| PCM 1222 | G5901 | *H. alvei* | KX117095 |
| PCM 1223 | G5897 | *H. alvei* | KX117096 |
| PCM 1224 | G5928 | *H. alvei* | KX117097 |
