# Supplementary material for: Genetic Diversity of O-Antigens in Hafnia alvei and the Development of a Suspension Array for Serotype Detection
Source: PLoS One. 2016 May 12;11(5):e0155115. doi: 10.1371/journal.pone.0155115 (PMC4869667; doi:10.1371/journal.pone.0155115)
Supplement: S2 Table — (DOCX) [file pone.0155115.s004.docx]

**Table S2. The *wzx* and *wzy* forms**

| **Strains** | **HGs of *wzx*** | **HGs of *wzy*** |
| --- | --- | --- |
| PCM 1188 | *wzx_1* | *wzy_1* |
| PCM 1189 | *wzx_2* | *wzy_2* |
| PCM 1191 | *wzx_3* | *wzy_3* |
| PCM 1192 | *wzx_4* | *wzy_4* |
| PCM 1194 | *wzx_5* | *wzy_5* |
| PCM 1196 | *wzx_6* | *wzy_6* |
| PCM 1198 | *wzx_7* | *wzy_7* |
| PCM 1202 | *wzx_8* | *wzy_8* |
| PCM 1204 | *wzx_9* | *wzy_9* |
| PCM 1209 | *wzx_10* | *wzy_10* |
| PCM 1210 | *wzx_11* | *wzy_11* |
| PCM 1211 | *wzx_12* | *wzy_12* |
| PCM 1212 | *wzx_13* | *wzy_13* |
| PCM 1214 | *wzx_14* | *wzy_14* |
| PCM 1216 | *wzx_15* | *wzy_15* |
| PCM 1218 | *wzx_16* | *wzy_16* |
| PCM 1220 | *wzx_17* | *wzy_17* |
| PCM 1221 | *wzx_18* | *wzy_18* |
| PCM 1222 | *wzx_19* | *wzy_19* |
| PCM 1223 | *wzx_20* | *wzy_20* |
| PCM 1224 | *wzx_21* | *wzy_21* |
