# Supplementary material for: Genetic Diversity of O-Antigens in Hafnia alvei and the Development of a Suspension Array for Serotype Detection
Source: PLoS One. 2016 May 12;11(5):e0155115. doi: 10.1371/journal.pone.0155115 (PMC4869667; doi:10.1371/journal.pone.0155115)
Supplement: S3 Table — (DOCX) [file pone.0155115.s005.docx]

**Table S3. The primers used in this study**

| **Strains** | **Lab. primer Number** | **Forward or Reverse** | **Primer** | **TM Value** |
| --- | --- | --- | --- | --- |
| PCM1223 | wl72741 | Forward | 5' TGCTATCTAGTGTGCTTGTTTTG 3' | 55.8 |
|  | wl72742 | Reverse | 5' CATTGACGGTGCCAAATT 3' | 54.4 |
| PCM1221 | wl72743 | Forward | 5' ATTCAAATTGCCTCACATTATT 3' | 54 |
|  | wl72744 | Reverse | 5' CCAACTGTCGATAGTGTGCC 3' | 56 |
| PCM1189 | wl72747 | Forward | 5' GTGTTAAATCGATTTAACGAATTA 3' | 54.3 |
|  | wl72748 | Reverse | 5' ACATCAAACGTTAACACAGAGTAA 3' | 54.4 |
| PCM1191 | wl72751 | Forward | 5' ATGATAGACCCATACAAATTTTAAG 3' | 54.7 |
|  | wl72752 | Reverse | 5' ACTCAGCCCGCACGTT 3' | 54.3 |
| PCM1192 | wl72753 | Forward | 5' TTAATCTTGTGCTATGTCTTTTATG 3' | 54.3 |
|  | wl72754 | Reverse | 5' GCCAATCGCGCTATACAA 3' | 55.5 |
| PCM1194 | wl72755 | Forward | 5' TGAGAAGTTGACTCCTATGCC 3' | 54.4 |
|  | wl72756 | Reverse | 5' TTTTGTTCTCTATGATTGTACAATCT 3' | 55 |
| PCM1196 | wl72757 | Forward | 5' AGGAAATCCTGGGGAAATT 3' | 55.2 |
|  | wl72758 | Reverse | 5' CGTAGCGCTTAATGTGGG 3' | 55 |
| PCM1198 | wl72759 | Forward | 5' TTTGCAACATACTCTTTCTTAACA 3' | 55 |
|  | wl72760 | Reverse | 5' AATGCGCAAGAGAACCAA 3' | 54.5 |
| PCM1202 | wl72761 | Forward | 5' AATGGTGCGTTAGCATCTAAA 3' | 55.7 |
|  | wl72762 | Reverse | 5' GATCCAACCACGGAAGAGAT 3' | 56.1 |
| PCM1209 | wl72765 | Forward | 5' TCAAGGCAGTCGCTGTCTA 3' | 54.6 |
|  | wl72766 | Reverse | 5' ATATCCAACGCACCTTCACT 3' | 54.5 |
| PCM1212 | wl72771 | Forward | 5' AGTAAACCCTGGGGCCA 3' | 55.4 |
|  | wl72772 | Reverse | 5' AGACAACGGCTACTTCACCA 3' | 55.4 |
| PCM1220 | wl73393 | Forward | 5' CAGGTTCGGTACTTACCTTTAGT 3' | 55 |
|  | wl73394 | Reverse | 5' TGAATCGATAAAAAATCAAAAAGA 3' | 55.6 |
| PCM1188 | wl73395 | Forward | 5' TCTTGATTTCGGAGGTTTCC 3' | 56.7 |
|  | wl73396 | Reverse | 5' TAGAGCACACCACCTGTAATTAA 3' | 55.1 |
| PCM1222 | wl73397 | Forward | 5' ATATGCCGATAGAGCTGAAGTT 3' | 55.8 |
|  | wl73398 | Reverse | 5' GCCCAACGAGCACCAGTT 3' | 58.8 |
| PCM1204 | wl73399 | Forward | 5' CCATGGGCATTCGAGTTTA 3' | 56.7 |
|  | wl73400 | Reverse | 5' AACAGACCAACTCCGCTAAAT 3' | 56 |
| PCM1210 | wl73401 | Forward | 5' GTTGGTCTTATCTGCGCTTATG 3' | 57.3 |
|  | wl73402 | Reverse | 5' GAAACAAACAGGCACCAGATA 3' | 55.3 |
| PCM1211 | wl73403 | Forward | 5' TAACTCTTTGGCTGGTTTGAAT 3' | 56.5 |
|  | wl73404 | Reverse | 5' CATATATTGAATTCAATCTAACATACCT 3' | 56 |
| PCM1214 | wl73405 | Forward | 5' TGGGAATGTTGAAACTTTTGG 3' | 57.8 |
|  | wl73406 | Reverse | 5' AAATACAGCTGTAATAGCGGATAT 3' | 55.3 |
| PCM1216 | wl73407 | Forward | 5' CTTTACGCTTAGTCCAGGAGG 3' | 56.5 |
|  | wl73408 | Reverse | 5' AGAGTGTAACTCCATAGTCTCTGTAAA 3' | 55.8 |
| PCM1218 | wl73409 | Forward | 5' TATGCGGCTAGAACCTTTGAG 3' | 57.7 |
|  | wl73410 | Reverse | 5' TAGGCCAGTAACTGTATATGTCAAA 3' | 56.4 |
| PCM1224 | wl73411 | Forward | 5' ATTGAGCAAGTAGGCTATGTTATT 3' | 55.1 |
|  | wl73412 | Reverse | 5' CGTATGTATCGATCATCGTGAA 3' | 56.3 |
