# Supplementary material for: Genetic Diversity of O-Antigens in Hafnia alvei and the Development of a Suspension Array for Serotype Detection
Source: PLoS One. 2016 May 12;11(5):e0155115. doi: 10.1371/journal.pone.0155115 (PMC4869667; doi:10.1371/journal.pone.0155115)
Supplement: S4 Table — (DOCX) [file pone.0155115.s006.docx]

**Table S4. The probe used in this study**

| **Strains** | **Lab. probe Number** | **Probe** | **Tm Value** |
| --- | --- | --- | --- |
| PCM1188 | OA5815 | 5' TTAGGGTCTCGAGCTTCATTTATACT 3' | 60 |
| PCM1189 | OA5816 | 5' TCCGTGAATACCACAGAACCAAT 3' | 60 |
| PCM1191 | OA6123 | 5' GTTACGCCAGCCACCTCTTCA 3' | 62.1 |
| PCM1192 | OA5819 | 5' TCTCAGATAACATGGTTCCTATTTTATC 3' | 59.5 |
| PCM1194 | OA5820 | 5' GCGCAACGGCAGTGTCA 3' | 59.9 |
| PCM1196 | OA5821 | 5' ATCTGTGGTCAGGGTGCTTTTT 3' | 60.3 |
| PCM1198 | OA5822 | 5' ATCGAGTCAGGCAGCTTTTAGC 3' | 60.8 |
| PCM1202 | OA5823 | 5' TAGGGCGACTAGCTGATGATTATT 3' | 59.8 |
| PCM1204 | OA5824 | 5' ACAGCCTCAACTGACCAATTAAGT 3' | 59.3 |
| PCM1209 | OA5825 | 5' TGTTCCCTCGGGTGAAATCA 3' | 60.8 |
| PCM1210 | OA6125 | 5' ATAAGTCCAAATGGACATACTTGGTAG 3' | 60 |
| PCM1211 | OA6126 | 5' AGGCTTGTAGGTAAAGTGAATGGC 3' | 61.3 |
| PCM1212 | OA5828 | 5' TGTGTGACCATCGCTGCTATTC 3' | 61 |
| PCM1214 | OA6127 | 5' TGGTAGAGTGGAAACACGTACATTT 3' | 60.1 |
| PCM1216 | OA6128 | 5' ATGTGGCTACGAAATTCCGGA 3' | 62 |
| PCM1218 | OA6129 | 5' CTGACTCTGGGCGTGTAATTAGAG 3' | 60.3 |
| PCM1220 | OA6121 | 5' TTCCTCGGTAGATACGGCACAA 3' | 62.2 |
| PCM1221 | OA5814 | 5' TTCCTACCAGAGCCTGCAGTG 3' | 60.3 |
| PCM1222 | OA5817 | 5' TTCTATTGATAGAATAATTATCCAT 3' | 59.7 |
| PCM1223 | OA5813 | 5' AGGAAAGCGAGCGACTTGC 3' | 60.5 |
| PCM1224 | OA6130 | 5' CTGCTTACCCATTGGTTATTTTTTAC 3' | 60.7 |
