# Supplementary material for: Genetic Diversity of O-Antigens in Hafnia alvei and the Development of a Suspension Array for Serotype Detection
Source: PLoS One. 2016 May 12;11(5):e0155115. doi: 10.1371/journal.pone.0155115 (PMC4869667; doi:10.1371/journal.pone.0155115)
Supplement: S5 Table — (DOCX) [file pone.0155115.s007.docx]

**Table S5. The GT names and HGs**

| **Strains** | **GT Names** | **Homology Groups** | **Fuctions** |
| --- | --- | --- | --- |
| PCM1188 | *whaA* | HG06 |  |
|  | *whaB* | HG08 | Gal-(β1-3)-GlcNAc |
|  | *whaC* | HG09 | Man-(α1-4)-Gal |
|  | *whaD* | HG40 |  |
| PCM1189 | *whaE* | HG04 |  |
|  | *whaF* | HG08 |  |
|  | *whaG* | HG10 |  |
|  | *whaH* | HG11 |  |
| PCM1191 | *whaI* | HG05 |  |
|  | *whaJ* | HG08 | Gal-(β1-3)-GalNAc |
|  | *whaK* | HG13 |  |
|  | *whaL* | HG14 |  |
|  | *whaM* | HG31 |  |
| PCM1192 | *whaN* | HG14 |  |
|  | *whaO* | HG15 | L-Rha-(β1-4)- |
|  | *whaP* | HG16 |  |
|  | *whaQ* | HG17 |  |
|  | *whaR* | HG31 |  |
| PCM1194 | *whaS* | HG01 |  |
|  | *whaT* | HG30 |  |
|  | *whaU* | HG35 |  |
|  | *whaV* | HG49 |  |
|  | *whaW* | HG50 |  |
| PCM1196 | *whaX* | HG03 |  |
|  | *whaY* | HG04 |  |
|  | *whaZ* | HG14 |  |
|  | *whbA* | HG18 |  |
| PCM1198 | *whbB* | HG06 |  |
|  | *whbC* | HG08 |  |
|  | *whbD* | HG32 |  |
|  | *whbE* | HG33 |  |
| PCM1202 | *whbF* | HG05 |  |
|  | *whbG* | HG08 |  |
|  | *whbH* | HG13 |  |
|  | *whbI* | HG29 |  |
| PCM1204 | *whbK* | HG04 |  |
|  | *whbL* | HG06 |  |
|  | *whbM* | HG06 |  |
|  | *whbN* | HG08 |  |
|  | *whbO* | HG09 |  |
| PCM1209 | *whbP* | HG14 |  |
|  | *whbQ* | HG19 |  |
|  | *whbR* | HG20 |  |
|  | *whbS* | HG21 |  |
| PCM1210 | *whbT* | HG06 |  |
|  | *whaB* | HG08 | Gal-(β1-3)-GlcNAc |
|  | *whbU* | HG15 | L-Rha-(β1-4)- |
|  | *whbV* | HG24 |  |
| PCM1211 | *whbW* | HG03 |  |
|  | *whbX* | HG06 | GlcNAc-(α1-4)-GalNAc |
|  | *whbY* | HG14 |  |
|  | *whbZ* | HG25 |  |
| PCM1212 | *whcA* | HG03 |  |
|  | *whcB* | HG13 |  |
|  | *whcC* | HG36 |  |
| PCM1214 | *whcD* | HG05 |  |
|  | *whcE* | HG14 |  |
|  | *whcF* | HG38 |  |
|  | *whcG* | HG39 |  |
| PCM1216 | *whcH* | HG11 |  |
|  | *whcI* | HG22 |  |
|  | *whcJ* | HG22 |  |
|  | *whcK* | HG23 |  |
| PCM1218 | *whcL* | HG03 |  |
|  | *whcM* | HG08 |  |
|  | *whcN* | HG17 |  |
|  | *whcO* | HG37 |  |
|  | *whcP* | HG42 |  |
| PCM1220 | *whcQ* | HG02 |  |
|  | *whcR* | HG03 | Gal-(α1-3)-GlcNAc |
|  | *whcS* | HG04 |  |
|  | *whcT* | HG05 | Gro-(1-P-6)-Glc |
|  | *whcV* | HG01 |  |
| PCM1221 | *whcW* | HG04 |  |
|  | *whcX* | HG06 |  |
|  | *whcY* | HG07 |  |
|  | *whcZ* | HG49 | Glc-(α1-P-6)-GlcN(R3Hb) |
| PCM1222 | *whcR* | HG03 | Gal-(α1-3)-GlcNAc |
|  | *whdB* | HG12 |  |
|  | *whdC* | HG37 |  |
| PCM1223 | *whdD* | HG08 |  |
|  | *whdE* | HG14 |  |
|  | *whdF* | HG27 |  |
|  | *whdG* | HG28 |  |
| PCM1224 | *whbX* | HG06 | GlcNAc-(α1-4)-GalNAc |
|  | *whaJ* | HG08 | Gal-(β1-3)-GalNAc |
|  | *whdH* | HG24 |  |
|  | *whdI* | HG26 |  |
